# Physical Activity for Health and Wellness

**DOI:** 10.3390/ijerph18157823

**Published:** 2021-07-23

**Authors:** Emanuela Gualdi-Russo, Luciana Zaccagni

**Affiliations:** 1 Department of Neuroscience and Rehabilitation, Faculty of Medicine, Pharmacy and Prevention, University of Ferrara, 44121 Ferrara, Italy; luciana.zaccagni@unife.it; 2 Center for Exercise Science and Sport, University of Ferrara, 44121 Ferrara, Italy

## 1. Introduction

Regular physical activity (PA) is both a preventive measure and a cure for non-communicable diseases (NCDs). Moreover, PA improves mental health, quality of life, and well-being [1]. Conversely, physical inactivity and sedentary lifestyles have negative impacts on individuals, families, and society, as evidenced in particular by the spread of the obesity epidemic [2,3,4,5,6].

PA has proven to be a low-cost alternative for the treatment and prevention of disease. Therefore, interventions to prevent avoidable diseases by increasing the proportion of physically active people are fundamental. 

The Special Issue “Physical Activity, Wellness and Health: Challenges, Benefits and Strategies” was intended to collect research articles on anthropometric determinants of health and performance, PA and healthy habits, exercise and diet, exercise and body composition, interventions to promote PA for people of all ages, strategies for the implementation of an active life, and the beneficial effects of exercise on metabolic syndrome. Finally, 20 articles covering a wide range of information were published, indicating the interest generated by this call. Below we will provide a summary of the main contents of this Special Issue, highlighting proposals for future research that potentially contribute to the health benefits of being physically active.

Topics included in this Special Issue fall mainly into the following three areas: anthropometry, health, and sport; health benefits of exercise; population studies and strategies for an active life.

## 2. Anthropometry, Health, and Sport

Anthropometric characteristics are important factors of a person’s physical performance and health status. Four studies included in this Special Issue evaluated the contribution of these variables. Matias et al. [7] found that phase angle derived from bioelectrical impedance spectroscopy is predictive of maximal isometric forearm strength in cancer patients. Its relevance as a clinical indicator of disease-related function in breast cancer survivors was suggested. Handgrip strength was particularly influenced by body composition parameters and handedness according to Zaccagni et al. [8], so much so that the authors recommended it as a proxy for unhealthy conditions with impaired muscle mass, taking into account laterality. Further research should also provide evidence for the effectiveness and clinical relevance of hand strength testing in the assessment and prediction of critical health conditions. Barbieri et al. [9] investigated the efficacy and accuracy of a data mining methodology in predicting cardiovascular risk based on anthropometric, demographic, and biomedical data from a very large sample of the population involved in competitive sports practice. The procedure was conducted using a decision tree and logistic regression to classify individuals as at-risk or not. In addition, the authors used the receiver operating characteristic curve to assess classification performance, achieving satisfactory results. The fourth study by Rinaldo et al. [10] departs from the previous themes to deal with injuries that can occur in sporting activities, focusing on the relationship between anthropometric traits and injury occurrence. Their findings pointed out that an increased body mass index, decreased calf muscle area, and being closer to the age of peak height velocity are significant risk factors for injuries in elite soccer players aged 9–13 years. Consistently with these findings, the authors claim that body composition and anthropometric characteristics should be monitored to reduce the risk of injury in young soccer players. Furthermore, training programs must be adapted to both the chronological age and the maturity status of the players.

## 3. Health Benefits of Exercise

PA contributes to preventing and treating a wide range of NCDs and can improve mental health, while also enhancing the quality of life and well-being. A total of seven studies in the Special Issue were conducted in this area.

Two studies concern, in particular, the implications of regular exercise for disease prevention and treatment. Kanai et al. [11] reported that the health utility score was 0.77 in stroke survivors and was associated with the number of steps; the more stroke survivors walked, the higher their health utility score. Turning to multiple sclerosis, it is well known that physical inactivity reduces cardiorespiratory capacity, promotes physical deconditioning, and leads to comorbidities such as obesity, metabolic syndrome, and osteoporosis. In this field, Pau et al. [12] examined possible sex-related differences in the amount and intensity of PA performed by people with multiple sclerosis and showed that the pattern for women was characterized by greater sedentariness and less activity of light intensity than for men. Both studies [11,12] quantitatively assessed PA (moderate-to-vigorous physical activity, MVPA) using accelerometers.

Five studies focused, in particular, on mental health and PA. PA promotes different kinds of positive psychological responses. Regular exercise has a beneficial impact on depression and anxiety. It reduces stress and improves overall well-being. The first study starts from the evidence that poor sleep quality, common in young people, increases the risk of morbidity and mortality. In this area, Zhai et al. [13] highlighted that regular PA can improve poor sleep quality among college students. PA could enhance sleep by helping individuals cope with stress, indicating that stress management could be a non-pharmaceutical treatment for sleep improvement. Considering the mental health of young people, Usán Supervía et al. [14] examined the relationships between the constructs of goal orientations, emotional intelligence, and burnout in high school students. The authors outlined that the psychological profile arising from these features could be important for academic performance and school participation. Bíró et al. [15] examined gender, as a socio-economic determinant of health, by testing the validity of the biopsychosocial model of health with a limited life course perspective on a very large sample of students from Hungarian universities and colleges. Their findings suggested that determinants of male health included fewer variables focused on physical activity, and were less influenced by social relationships, in contrast to female health, which was influenced by age and social support. Kim and Ahn [16] showed that exercise participation for six weeks led to positive changes in the self-esteem and mental health of college students. In a narrative review, Belvederi Murri et al. [17] investigated the beneficial effects of PA on depressed populations. A specific public health problem is the premature mortality of depressed individuals. This is mainly caused by increased cardiovascular risk, as depression leads to the development or exacerbation of unhealthy lifestyles. According to their findings, PA can reduce depression severity and directly address cardiovascular risk factors. In the field of public health, the development and dissemination of initiatives promoting exercise-based interventions in depressed populations are recommended, focusing on their cost-effectiveness.

## 4. Population Studies and Strategies for an Active Life Implementation

Nine articles in the Special Issue deal with this topic.

Two studies took into account the multiple negative effects of physical inactivity on health and the factors involved. In a South African adult population, Chifaku et al. [18] assessed the levels and correlates of PA. They found that gender, marital status, and health awareness were significant predictors, pointing out a high prevalence of insufficient PA in some vulnerable groups, particularly the elderly and obese, and a general lack of participation in sports and recreational activities. As PA plays a fundamental role in the process of growth and development, Baqal et al. [19] analyzed data from a national study, “Jeeluna”, on a large sample of adolescents living in the Kingdom of Saudi Arabia. The authors found that 67% of adolescents who did not exercise led a sedentary lifestyle. Males and adolescents aged 10–14 years were significantly more likely to engage in PA than females and adolescents aged 15–19 years. Among the factors contributing to high rates of inactivity among adolescents, the authors include the lack of PA programs in schools, hot weather conditions, poor family and peer support, and socio-cultural barriers, which have a particular impact on girls.

Despite the known benefits of regular PA, there is a high percentage of physically inactive adults worldwide. Increased national attention on PA as a tool for health promotion and disease prevention is therefore required [20]. Five studies in this Special Issue examine different approaches and strategies that aim to increase PA. The first article, by Potter et al. [21], is a pilot study on activities that naturally involve PA, considering a stealth health approach to increase PA among inactive dog owners. The approach tested in this study showed that dog obedience training could have, as a side effect, a positive impact on both PA and sedentary behavior among dog owners; dog owners are induced to walk more and sit less. Given the large number of dog owners, this new approach to promoting PA may have a significant impact on public health and merits further investigation. In Latin America, the prevalence of obesity and overweight is increasing in all countries, despite the efforts of governments to promote healthy lifestyles. In this context, Farías [22] analyzed which emotions out of fear and hope are most effective in stimulating individuals to make health-related decisions, showing that these appeals in health advertisements do not have any main effect on PA intention, although this effect is positively moderated by perceived body weight and past healthy eating behavior, and is negatively moderated by subjective norms in diet and exercise. Another study conducted by Shi et al. [23] on university students indicated that the combination of insufficient physical activity levels with mobile phone addiction is significantly linked to high levels of irrational procrastination. To improve efficiency and reduce irrational procrastination, it would be necessary to increase physical activity and reducing mobile phone addiction. A systematic review by Zaccagni et al. [24] reported the consequences on physical activity and health of the general lockdown implemented in Italy from March to May 2020 due to the COVID-19 pandemic. Their analysis of 23 studies showed that there has been a general reduction in PA and unhealthy dietary habits as a result of this lockdown in Italy, with a deterioration of the health status in both the general population and people with chronic diseases. According to the authors, individual outdoor exercise should be promoted, especially during daylight hours, while maintaining physical distance in the case of another lockdown to contain current and future pandemics. Particularly in older people, sedentary behavior is a serious public health problem. Monteagudo et al. [25] examined the impact of overground walking interval training in sedentary older adults by comparing two different dose distributions during a longitudinal study. Both training protocols led to a significant overall improvement of physical function in older adults. As regards the strategy to be used in the elderly, Monteagudo showed that the bout length is not a determinant of the functional health effects associated with exercise; splitting a single exercise into two sets during the day can be beneficial for autonomy, agility, and health-related quality of life. In particular, the accumulative strategy is to be recommended when health-related quality of life is the main goal, whereas the continuous strategy is to be recommended when weakness may be a short- or medium-term threat.

The last two studies of this section concern the fitness sector and the spread of sports venues. The research of Moustakas et al. [26] aimed to define the drivers of change in the fitness sector and to identify the skills needed by the fitness workforce to navigate these changes. The main finding was that technology, health needs, and customer loyalty are critical drivers of change in the fitness industry. Fitness professionals must therefore respond by improving both their professional skills, especially in providing services for special populations, and their soft skills, stressing the particular importance of engaging with technology and having an understanding of specific health issues. Mainland China, one of the most populous upper-middle-income countries, also has to deal with a prevalence of NCDs and physical inactivity. Analyzing the relevant characteristics of sports venues associated with leisure-time PA in China, Wang et al. [27] identified the number and area of sports venues as the most important indicators. The number of sports venues, which increased between 2000 and 2013, is still comparatively small compared to the United States and Japan. The urban–rural gap in sports venues exemplifies just a few aspects of the ‘urban–rural dual structure’ in Chinese society.

## 5. Conclusions

The 20 manuscripts included differ in subject matter and methodologies applied, and we consider this variability to be an enrichment for the Special Issue. According to the previous subdivision, the studies included in this Special Issue dealt mainly with interventions to promote PA for persons of all age groups and implementation strategies for active living in different populations.

In general, the studies made important suggestions for planning targeted interventions for specific diseases, ages, or population groups, but also for providing guidelines for a healthy lifestyle, tailored to the requirements of individuals to achieve maximum effectiveness. PA interventions are needed to reduce the treatment costs of chronic morbidity that may result from a lower prevalence and better control of CVD and its risk factors. PA-based interventions have also been shown to be effective as additional interventions in mental health. In this respect, it should be emphasized that exercise is still underprescribed for depressed individuals. It is therefore important to eliminate the barriers that are currently restricting this prescription by clinicians.

The findings of several studies support the relevance of specific anthropometric variables as potential health indicators, suggesting that anthropometric characteristics and growth rates should be monitored in younger athletes. To improve clinical decision making by reducing the number of unnecessary examinations, the application of data mining to biomedical data, including anthropometric data, may be effective.

The importance to ensure the application of appropriate methodologies of measuring quantitative traits (PA, strength, body composition measurements, etc.) was often emphasized in the articles.

All of the studies support strategies to promote PA and reduce sedentary behavior among adolescents, adults, and the elderly. There is no doubt that regular exercise is beneficial to health, but the general population should be encouraged to engage in more of it.

With the support of all the contributing authors, we are confident that we have provided a significant contribution to the knowledge of the topic addressed in this Special Issue.

## Data Availability

Not applicable.
